# Deciduous Teeth; Their Importance; Their Preservation

**Published:** 1865-08

**Authors:** N. H. Tulloss


### DECIDUOUS TEETH; THEIR IMPORTANCE; THEIR PRESERVATION.

BY N. H. TULLOSS.

[Read before the Iowa State Dental Society, at Dubuque.]

It seems as though enough had been said on this subject to have unanimity of sentiment in favor of the preservation of these teeth. But my experience tells me that many self-styled dentists are ruthlessly destroying the mouths of innocent children.

Why wont those ignorant quacks learn better, or is it the "quarter" that blinds their judgment and conscience.

If the deciduous teeth had not been necessary to the healthy development of the physical constitution of the child, the Almighty would have created only the *permanent* ones. God makes nothing that is not useful.

Every physiologist knows that food received into the stomach without mastication is injurious to that organ. Want of the deciduous teeth is often a source of permanent dyspepsia, continuing life-long. Without these teeth language is imperfectly developed. Lisping is acquired which often lasts even into adult life, to the mortification of its subject. How *ashamed* a *young man* is with a lisping accent; he is the butt of his lady acquaintances; and if he is not blessed with extra common sense, will meet with contempt from his male acquaintances.

We all know what effect extraction has on the alveoli. Rapid absorbtion of the bone and contraction of the animal tissues take place. Here then commence many evils. Want of room for permanent teeth, which are generally larger than the ones they replace. Irregularity as a consequence surely followed by dental decay.

Parents fail to do their duty. They let their children grow up without the least care of their deciduous teeth. They decay of course, and finally ache; and then of course they visit a dentist to have them extracted. But they run against a stump if they come to me. I do not extract the poor little thing's teeth if I think I can save them. I kill the pulp, treat and fill with cheap material. And it is surprising how well children's teeth bear treatment. They are seldom troublesome after destroying the pulp and filling. I find, too, that the pulp is less sensitive than that in the permanent teeth.

If parents would teach their children to keep their teeth clean, and not allow them to disorder the digestive functions by over eating, too frequent or by improper food, they would save these innocent defendants weeks, months and even years of tooth ache.

Here are seven or eight years during which permanent teeth are taking the place of the deciduous ones. It is necessary for me to say that these first teeth being decayed, infect those they approximate.

We all know the evil results of decay in deciduous teeth, and the greater evil of extraction.

Why do we not, then, have the moral courage to say that we wont extract these teeth, and also labor by every means to induce parents to do justice by their children.

It is really a pleasure to work for children. They have so much patience Their courage and fortitude is remarkable. It is easily developed by kind encouraging words.

As to materials for temporary plugs; gold is unnecessary. Yet when parents are able and willing, I would use it. We ought to cultivate a habit and taste for gold in the teeth. It is of more importance than some may think. If a child has had its teeth filled with gold, you never need to expect, when "grown up," that any thing but gold will do for him or her, in dental operations.

Still tin foil makes a good filling in lateral and approximal cavities.

Hill's stopping can be used in the same situation when the teeth are to be retained but a year or two. But we must not trust too much to the tooth of replacement. Sometimes they never *come.* How often we see this in the upper canines. I possess my superior left deciduous canine to-day, and am in my fortieth year. If this class of temporary teeth decay, I would fill them with gold if allowed to. Lawrence's amalgam is very good for crown cavities.

Now, brethren, let us take an united stand on this subject of the importance of the preservation of the deciduous teeth.

I hope a resolution will be offered on this subject which will embody the sense of this society, and then let us have it published in the newspapers.

I am sorry, gentlemen, that circumstances are such that I cannot be with you at the present session of our society, (being the first that I have not attended since we organized,) but if not present in person, I will be in spirit.

